# Recent Advances and Prospects in the Research of Nascent Adhesions

**DOI:** 10.3389/fphys.2020.574371

**Published:** 2020-12-04

**Authors:** Bernd Henning Stumpf, Andreja Ambriović-Ristov, Aleksandra Radenovic, Ana-Sunčana Smith

**Affiliations:** ^1^PULS Group, Institute for Theoretical Physics, Interdisciplinary Center for Nanostructured Films, Friedrich-Alexander-Universität Erlangen-Nürnberg, Erlangen, Germany; ^2^Laboratory for Cell Biology and Signalling, Division of Molecular Biology, Ruđer Bošković Institute, Zagreb, Croatia; ^3^Laboratory of Nanoscale Biology, École Polytechnique Fédérale de Lausanne, Lausanne, Switzerland; ^4^Group for Computational Life Sciences, Division of Physical Chemistry, Ruđer Bošković Institute, Zagreb, Croatia

**Keywords:** nascent adhesions, focal adhesions, integrin activation, integrin clustering, superresolution microscopy, modeling

## Abstract

Nascent adhesions are submicron transient structures promoting the early adhesion of cells to the extracellular matrix. Nascent adhesions typically consist of several tens of integrins, and serve as platforms for the recruitment and activation of proteins to build mature focal adhesions. They are also associated with early stage signaling and the mechanoresponse. Despite their crucial role in sampling the local extracellular matrix, very little is known about the mechanism of their formation. Consequently, there is a strong scientific activity focused on elucidating the physical and biochemical foundation of their development and function. Precisely the results of this effort will be summarized in this article.

## 1. Introduction

**Figure 1:**
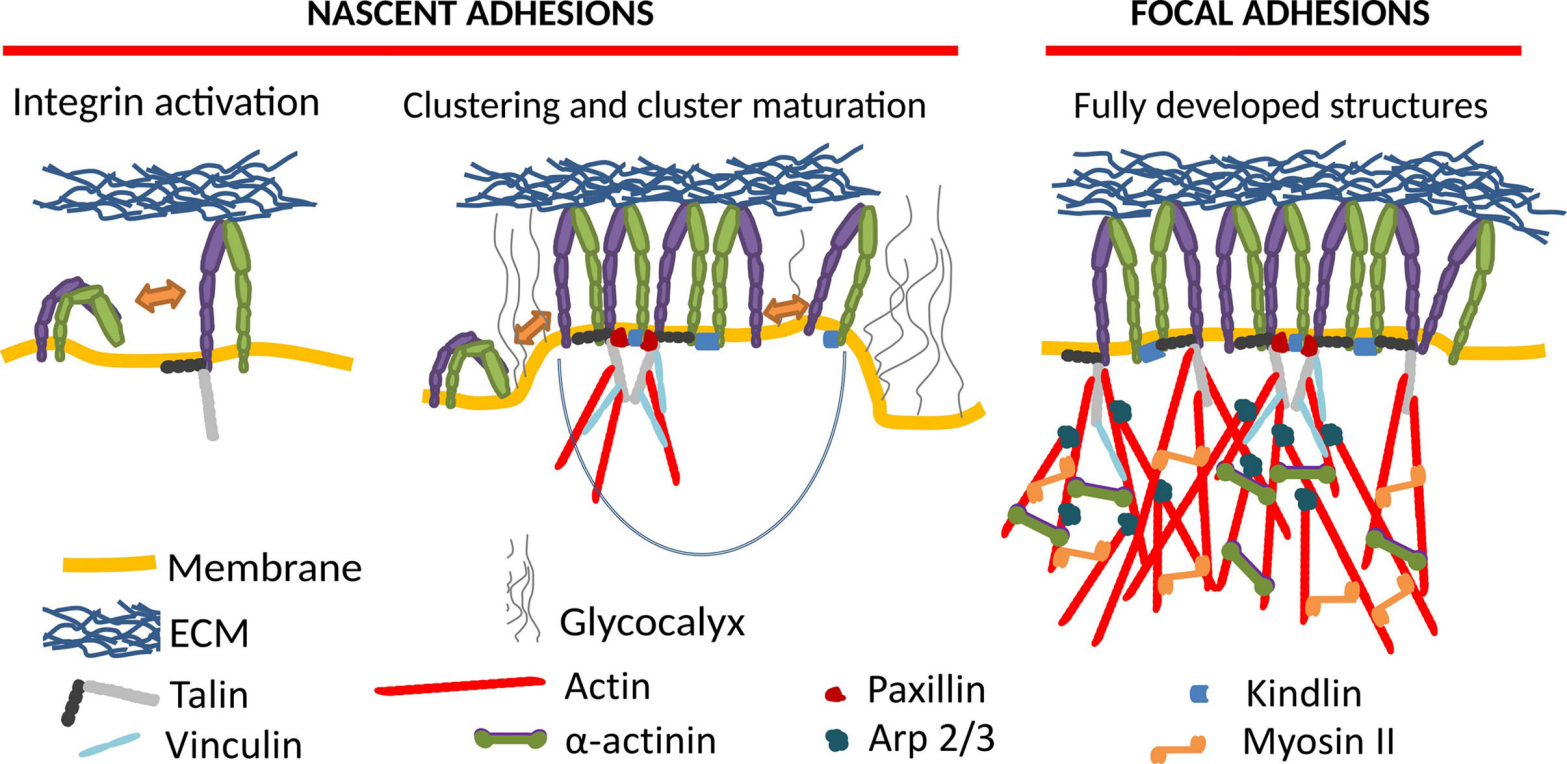
Formation of integrin mediated cell adhesion structures requires integrin activation **(left)**, which induces macromolecular clustering into nascent adhesions **(middle)**, which upon maturation grow into focal adhesions **(right)**. The key molecular players in this process are schematically represented as shown in the legend.

Integrin-mediated adhesion of cells and the associated mechanosensing is of monumental importance for the physiology of nearly any cell type (Kechagia et al., 2019; Samaržija et al., 2020). Upon integrin activation, it often proceeds through the maturation of nascent adhesions (NAs) to focal adhesions (FAs) (Figure 1), which are transient supramolecular assemblies, connecting a cell to the extracellular matrix or another cell. NAs typically contain around 50 integrins (Changede et al., 2015), and show a high turnover rate, with lifetimes of a bit over a minute (Choi et al., 2008). FAs, which arise upon the maturation of NAs by recruitment of numerous proteins to their cytoplasmic tails, form multimolecular integrin adhesion complexes. FAs are establishing the linkage between the extracellular matrix (ECM) and the actin cytoskeleton (Winograd-Katz et al., 2014). However, another cytoskeletal element, microtubules, also plays an important role in adhesion and regulates the turnover of adhesion sites (Bouchet et al., 2016; Chen et al., 2018).

While the FAs have been studied extensively in the last decades (Geiger et al., 2009; Parsons et al., 2010; Geiger and Yamada, 2011; Wehrle-Haller, 2012; Cooper and Giancotti, 2019; Green and Brown, 2019), the smaller characteristic size of ~100 nm diameter makes NAs significantly more elusive (Changede et al., 2015). Studies of NAs require single molecule localization microscopy (SMLM) techniques to image below the diffraction limit of conventional light microscopy, and, furthermore, need to account for the short characteristic lifetime of few minutes (Changede et al., 2015). The resulting scarcity of data associated with NAs makes the theoretical modeling very difficult.

In our current understanding, the NA formation follows three major steps (Sun et al., 2014). First, integrins activate, going from a state of low to high affinity, potentially through a conformational change. This can be induced by the binding of activating proteins like talin or kindlin (Cluzel et al., 2005; Humphries et al., 2007; Saltel et al., 2009; Ye et al., 2013; Ellis et al., 2014; Changede et al., 2015), or by binding to a ligand (Barczyk et al., 2010). In the second step, the integrins cluster into NAs, which show similar structures on substrates of different rigidities (Changede et al., 2015), and are not reliant on myosin II (MII) activity (Choi et al., 2008; Bachir et al., 2014; Oakes et al., 2018). Finally, these clusters are either disassembled, or they mature into FAs and possibly further into fibrillar adhesions.

Despite the efforts leading to our current understanding, the determinants of NA formation, turnover, or maturation, as well as their role in mechanosensing and signaling, are far from being fully resolved. However, the past two decades witnessed the emergence of several novel optical imaging techniques, technological advances in protein engineering and mass spectrometry analysis, as well as the expansion of theoretical modeling that now allow the investigation of protein organization of NAs at the nanoscale. Motivated by these perspectives, we here attempted to recapture recent advances in the field, while identifying open questions which we believe will be addressed in future research.

## 2. The Key Molecular Players in Nascent Adhesions

Nascent adhesion revolves around the formation of integrin-ligand contacts between the cell and the extracellular matrix. Integrins are cell adhesion proteins capable of sensing the mechanical properties of the cell environment and providing signals necessary for a number of cell functions including proliferation, and migration (Horton et al., 2015; Cooper and Giancotti, 2019; Green and Brown, 2019; Humphries et al., 2019; Michael and Parsons, 2020). In humans, this broad range of functionalities is maintained by 24 integrin heterodimers (Shimaoka and Springer, 2003) built from 18 α- and 8 β-subunits forming a headpiece and two legs (Xiong, 2001). Several combinations of α and β subunits forming a heterodimer are possible (Hynes, 2002; Campbell and Humphries, 2011). However, only integrins assembled as heterodimers in the endoplasmic reticulum are expressed on the cell surface. Therefore, the composition of the plasma membrane integrin repertoire cannot be reliably predicted by the mRNA expression levels (Hynes, 2002) of integrin subunits. Integrin expression can be regulated by modulating their internalization and recycling, which contributes to the dynamic remodeling of adhesion (Moreno-Layseca et al., 2019).

Most integrins promiscuously bind to several ligands. Furthermore, their interactions are also extremely redundant, as different integrins bind to the same ligand (Humphries et al., 2006). Therefore, the 24 heterodimers are broadly categorized by their specificity to the ECM as (i) Arg-Gly-Asp receptors, binding to fibronectin, fibrinogen, and thrombospondin, (ii) laminin receptors, (iii) collagen receptors, and finally (iv) leukocyte-specific receptors binding to different cell surface receptors such as intercellular adhesion molecule and some ECM proteins (Humphries et al., 2006; Takada et al., 2007; Barczyk et al., 2010). Additional ligands relevant in the immunological context are the intercellular adhesion molecules, immunoglobulin superfamily members present on inflamed endothelium, and antigen-presenting cells. From the four groups, however, only the first three are considered to contribute to NAs.

Integrin binding and clustering is promoted by, and contributes to the formation of multimolecular integrin adhesion complexes involving up to 2,400 proteins together termed adhesome. The composition of integrin adhesion complexes have been first analyzed for cells seeded on fibronectin (Zaidel-Bar et al., 2007; Kuo et al., 2011; Schiller et al., 2011; Byron et al., 2015; Jones et al., 2015), identifying 60 core proteins involved in the fibronectin-induced meta adhesome, termed the consensus adhesome (Horton et al., 2015). One particularly important family of molecules within the consensus adhesome are the so-called adaptor proteins which bind to the cytoplasmic tails of integrins and bridge to the actin-based cytoskeleton. There are four potential axes that link integrins to actin, all implicated in different stages of NA formation, namely (i) integrin-linked kinase-particularly interesting new cysteine-histidine rich protein-1-kindlin, (ii) focal adhesion kinase (FAK)-paxillin, (iii) talin-vinculin and (iv) α-actinin-zyxin-vasodilator-stimulated phosphoprotein (Winograd-Katz et al., 2014; Horton et al., 2015, 2016; Humphries et al., 2019). Interestingly, some adaptors, such as talin, also coordinate the microtubule cytoskeleton at adhesion sites through the interaction with KANK proteins (KN motif and ankyrin repeat-containing proteins) (Bouchet et al., 2016; Sun et al., 2016; Chen et al., 2018; Paradžik et al., 2020), which was shown to stimulate FA turnover (Stehbens and Wittmann, 2012). How early this connection is established and the implication to NAs is still unresolved.

A lot of detail regarding integrin structure and interactions with adaptor proteins is obtained from molecular dynamics simulations, which starting with the seminal works on conformational changes in activation (Puklin-Faucher et al., 2006), addressed integrin unfolding (Chen et al., 2011), differences in integrin transmembrane domains (TMDs) (Pagani and Gohlke, 2018), talin-integrin interactions also regarding the surrounding lipids (Kalli et al., 2017), and interactions with other proteins (Shams and Mofrad, 2017).

Most integrin-related research, nevertheless, involves studies of mature adhesions, particularly in the context of the relation between the integrin adhesion and the cell physiology. This relation revolves around signaling involves a number of kinases, phosphatases, guanine nucleotide exchange factors, GTPase activating proteins, and GTPases (Horton et al., 2016). From the perspective of NAs, they so far have been discussed in the context of the physiology of the FAs. However, with the recently initiated debate that NAs may themselves act as signaling platforms, new perspectives in targeting NA-associated processes emerge. However, harnessing these possibilities requires detailed knowledge of the sensory role of NAs, their dynamic behavior, and their regulation, which are all still poorly understood.

## 3. The Onset of Nascent Adhesion: Integrin Activation

Activation is the first step in the formation of NAs and is associated both with a change in integrin affinity and the binding of integrins to extracellular ligands (Calderwood, 2004). Activation as a term is also used to signify the switch to the extended-open (EO) conformation, which is, with the bent-closed (BC) and the extended-closed states, one of three major integrin conformations (Luo et al., 2007). All three conformations may be specific to one or more ligands, with affinity being conformation dependent (Wang et al., 2018), Often though, the activated EO state is the one with the highest binding affinity (Li J. et al., 2017). For example, prior to activation, the BC state is the most common conformation of α5β1 in the K562 chronic myelogenous leukemia cell line, making up for around 99.76% of the population (Li J. et al., 2017). Simultaneously, the extended-closed and EO states contribute with 0.09 and 0.15%, respectively. However, α5β1 and α4β1 in the EO state have a 4,000–6,000 fold and a 600–800 fold higher affinity for a ligand compared to the BC state (Li and Springer, 2018). Notably, these affinities are measured for ligands in solution, where they do not induce integrin clustering (Cluzel et al., 2005).

**Figure 2:**
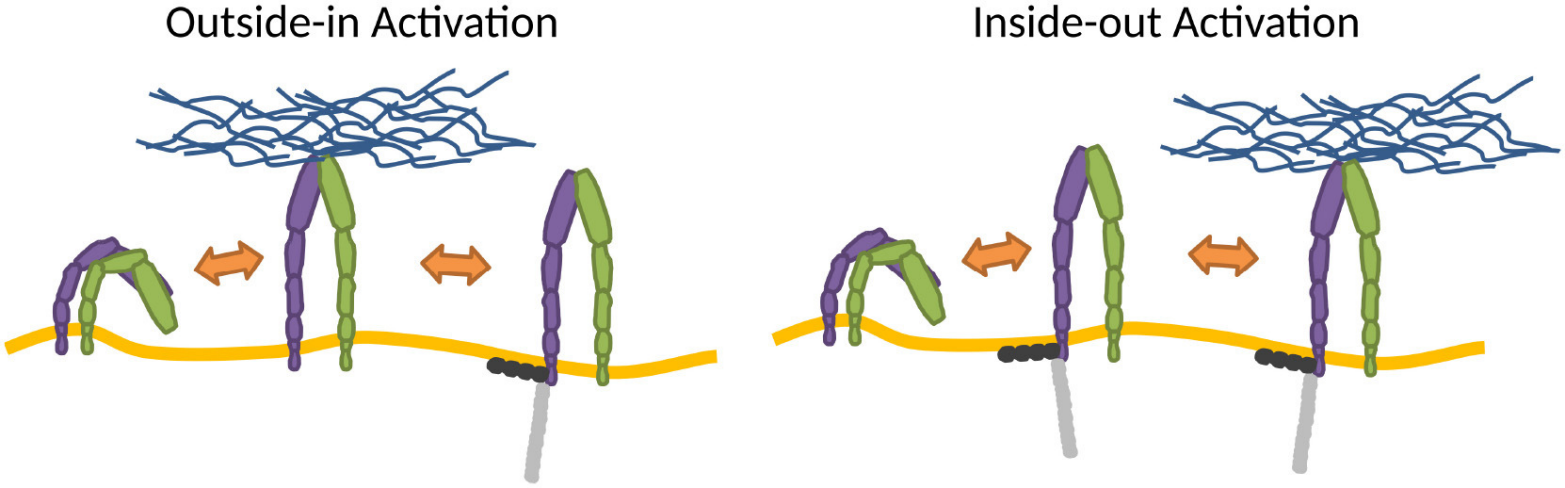
Mechanisms of integrin activation. In the outside-in activation, extracellular factors activate the integrin (Barczyk et al., 2010; Park and Goda, 2016), subsequently stabilized by binding adaptor proteins to the cytoplasmic tail. In the inside-out activation, binding of cytoplasmic factors induces activation (Ye et al., 2010; Calderwood et al., 2013), which primes the integrin for ECM binding. Integrins also stochastically switch between conformations.

The changes of conformation may be introduced by thermodynamic fluctuations of the integrin (Sun et al., 2019) or strong membrane deformations (Gingras and Ginsberg, 2020), but the switch is most often induced by the very association of integrins with ligands, adaptor proteins or Mn^2+^. However, Mn^2+^ may induce integrin conformations that can be different from physiological ones (Ye et al., 2012). In the cellular environment, the process within which integrins adopt the high affinity state is cast into two major activation models (Wang et al., 2018), the so-called outside-in, where the activation results from binding to extracellular ligands (Barczyk et al., 2010; Park and Goda, 2016), and inside-out, induced by cytoplasmic factors such as adaptor proteins (Ye et al., 2010; Calderwood et al., 2013), for example, by talin (Cluzel et al., 2005; Saltel et al., 2009; Park and Goda, 2016) (Figure 2).

A number of cell-related studied addressed inside-out activation. It is now established that already talin head domain is sufficient to activate integrins (Calderwood et al., 1999) and synergizes with kindlin (Ma et al., 2008; Bledzka et al., 2012; Calderwood et al., 2013). This combination may promote binding to multivalent ligands (Ye et al., 2013). However, there seems to be a competition between talin and kindlin, as the overexpression of kindlin-1 and kindlin-2 can both enhance and reduce integrin activation by talin head domain, depending on the integrin type (Harburger et al., 2009). In other cases, kindlin overexpression showed only a small effect compared to talin head domain (Shi et al., 2007; Ma et al., 2008; Ye et al., 2010, 2012, 2013).

**Figure 3:**
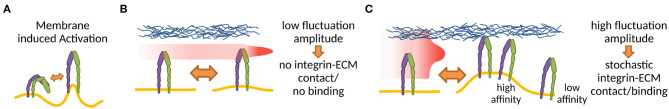
**(A)** Membrane deformations, for example through curvature or thickness changes, can disrupt integrin TMD interactions, increasing fluctuation induced affinity modulation (Gingras and Ginsberg, 2020). **(B,C)** The amount of interaction of an integrin with a ligand presenting counterpart effectively alters the integrin binding affinity (Schmidt et al., 2012; Fenz et al., 2017) in a membrane fluctuation and therefore also glycocalyx dependent manner. The red area in **(B,C)** shows the positional distribution of the integrin head as a result of membrane fluctuations. The small fluctuations in **(B)** result in no integrin-ECM contact, whereas the high fluctuations in **(C)** result in stochastic integrin-ECM contacts, allowing stochastic binding and creating high affinity regions around the initial bond.

Outside-in activation has been extensively studied both in cell and cell-mimetic systems. As pointed out already in the seventies, and then again by more recent work, the microenvironment of the plasma membrane and cell glycocalyx may play a significant role in the regulation of the receptor affinity for ligands (Bell, 1978; Dembo et al., 1988; Bihr et al., 2012; Fenz et al., 2017). Namely, the membrane, by its elasticity and fluctuations can induce switches from low to high affinity states (Figure 3), even without changing the actual conformation of the proteins binding the ligands (Fenz et al., 2011; Kim et al., 2020). This mechanism of regulation of affinity was originally suggested by theoretical modeling (Bihr et al., 2012), and was demonstrated for a variety of membrane associated ligand-receptor pairs (Bihr et al., 2015; Fenz et al., 2017). It relies on the modulation of membrane fluctuations in activating cells, and the expulsion of the glycocalyx. However, although this mechanism should be particularly relevant for the formation of NAs, its role for integrin binding remains to be shown explicitly. Preliminary hints for the role of this mechanism come from mimetic system where a liposome bind to a bilayer by the establishment of integrin-Arg-Gly-Asp bonds (Goennenwein et al., 2003; Smith et al., 2008; Streicher et al., 2009). The strength of adhesion and the number of formed integrin-Arg-Gly-Asp constructs depended sensitively on integrin density and mobility, hence the capacity to bind in proximity of an existing bond. This suggests that the membrane indeed mediates correlations and affects the integrin binding rates.

Binding of ligands allows for anchoring of integrins and the exertion of forces. Interestingly, recent work showed that even after the links with the cytoskeleton are fully established, most integrins existed in a state of near-mechanical equilibrium (Tan et al., 2020). This corroborates the idea that retractive actomyosin force is presumably acting only in FAs and not so much in NAs. However, the polymerization of actin in the cytoskeleton couples to the membrane fluctuations, which furthermore couples to the integrin bonds. Consequently, the unbinding rate of the receptor in a particular conformation is directly affected (Bihr et al., 2012), but also the free energy of BC, extended-closed and EO states may change. The importance of this effect was highlighted in the model of Li and Springer (2017), who find a sigmoidal dependence of activation probability with respect to the applied force, where activation here signifies binding of both ligand and adaptor protein. This resulted in full activation of all integrins over few pN for a wide range of adaptor protein concentrations, permitting a quick response to mechanical stimuli. Comparable behavior was found in fibroblasts, which reinforce early integrin adhesions ≤ 5 s under load by binding additional integrins (Strohmeyer et al., 2017). However, in this case the force was not applied to the integrin themselves but to the apical membrane of the cell. The response of this system can thus be similar to integrin decorated vesicles subject to pulling force, where the reinforcement was also observed as part of the thermodynamic response of the entire system (Smith et al., 2008).

Recent experiments revealed that upon mechanical stretching (2 to 5%) FA integrin β3 displacements closely followed the substrate's elastic displacements (Massou et al., 2020). Such behavior revealed that most stationary integrins inside and outside of FAs remained connected to fibronectin. Moreover, the same platform allowed to investigate whether proteins mediating a dynamic mechanical coupling of integrins to F-actin follow or deviate from integrins' elastic behavior (Massou et al., 2020). Massou et al. concluded that the spatiotemporal force fluctuation in FAs probably emerges from the heterogeneous tensional/connective states of proteins at the nanoscale (Massou et al., 2020).

The response of individual integrins to local force has to be considered also in the context of the catch-bond effect. Namely, unlike slip bonds, which subject to force show an increase in the unbinding rate (Bell, 1978), catch bonds are stabilized by force (Dembo et al., 1988). So far, in the case of integrins, both behaviors were found for different force regimes, which lead to the introduction of the term catch-slip bond. The latter was observed for example for α_5_β_1_ (Kong et al., 2009), α_4_β_1_ (Choi et al., 2014), and α_*L*_β_2_ (Chen et al., 2010). In the case of α_5_β_1_, catch bond formation seemed to involve the headpiece, but not integrin extension (Kong et al., 2009).

Other distinct mechanisms that, similarly to catch bonds, strengthen integrin attachments in a force dependent manner are cyclic mechanical reinforcement (Kong et al., 2013), and the so called dynamic catch (Fiore et al., 2014). In cyclic mechanical reinforcement, an increase of bond lifetime occurs over several loading-unloading cycles. In dynamic catch, the force response is regulated synergistically by the binding of an additional co-receptor to form a trimolecular complex with the integrin and the common ligand. In mimetic, actin free systems, cyclic application of force also resulted in bond-strengthening (Smith et al., 2008), which was shown to emerge from a thermodynamic response of the integrin ensemble. Application of a pulling force (Smith and Seifert, 2005) induced a regrouping of bonds from sparse configurations to clusters in which cooperative response is allowed (strengthening each bond on average), and a new thermodynamic state is established (Smith et al., 2008).

Despite all these efforts and insights, since the sequence of binding events in a cell is not yet fully established, activation is still heavily studied. As elaborated above, various possibilities for activation are available, but to what extend the cell relies on the different mechanisms remains to be clarified. Due to the stochastic nature of molecular binding, different types of activation could take place simultaneously on the cell surface. Upregulating certain molecular players then only changes the probability for observing a certain pathway. How is the whole process regulated? Does the cell choose to shift the balance toward a particular activation mechanism, and if so why and how are just some of the questions which will need to be answered in the future.

## 4. Formation of Integrin Clusters

**Figure 4:**
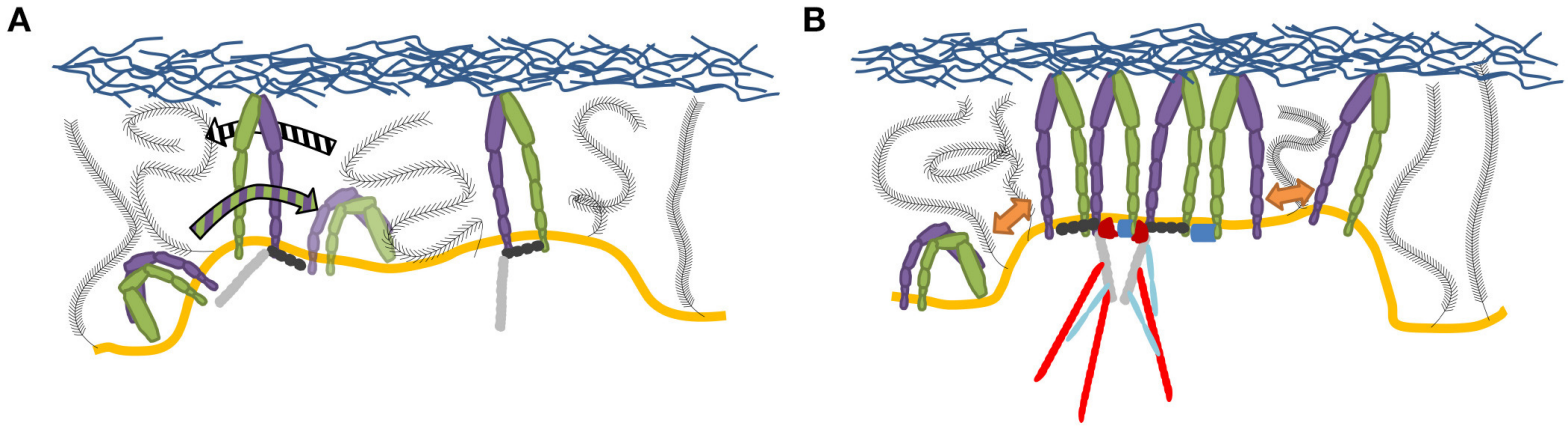
Current picture of integrin clustering. **(A)** Initial liganded integrins create a region with increased integrin binding probability, by membrane and glycocalyx deformation, which effectively attracts integrins while pushing out the glycocalyx. **(B)** Clustering is further amplified by multivalent ligands, dimerizing adaptor proteins, established scaffolding structures through cytoplasmic factors as well as TMD interactions (Gingras and Ginsberg, 2020).

**Figure 5:**
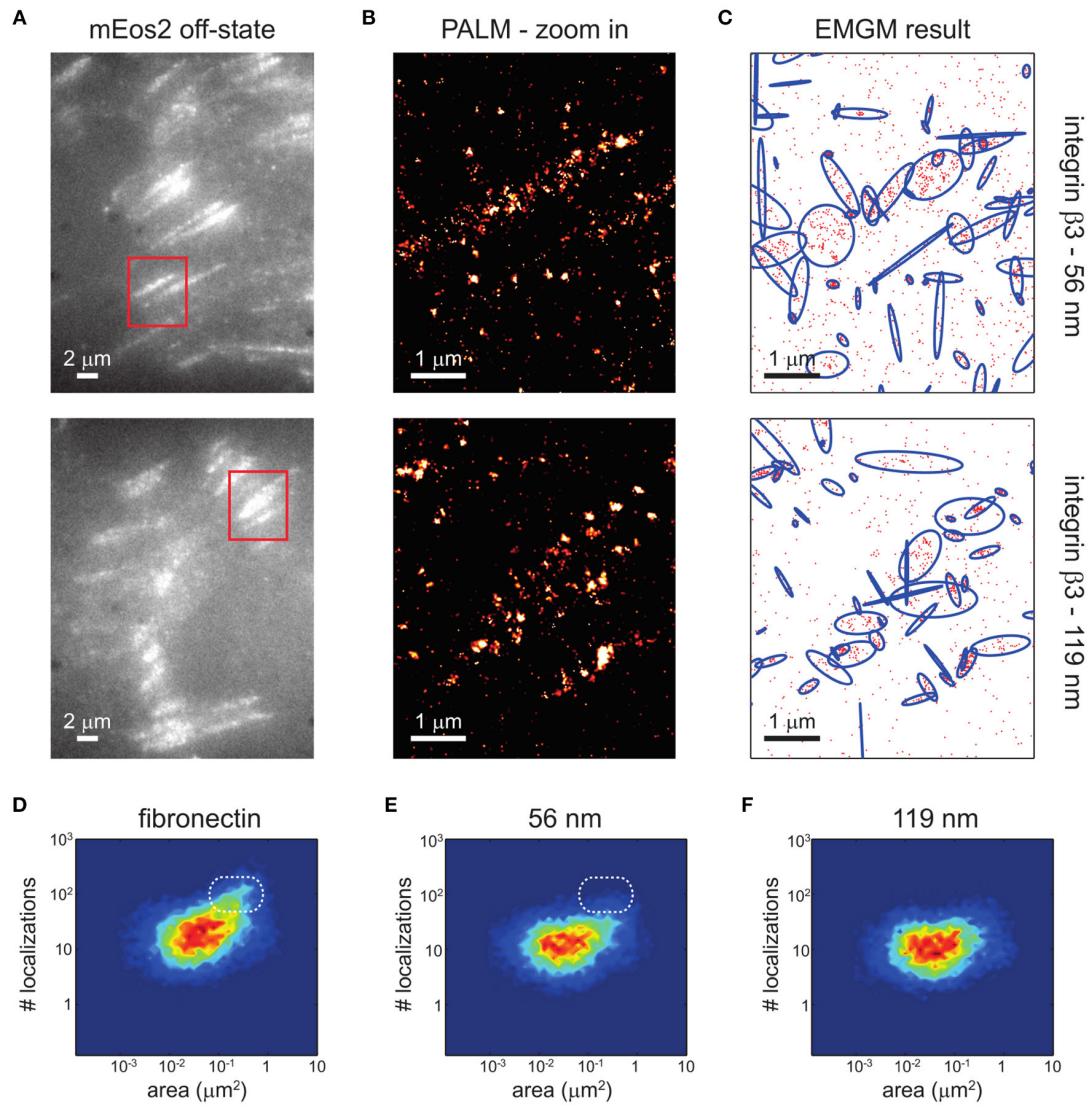
EMGM analysis of PALM data of integrin β3 on nanopatterned substrates. **(A)** Shown here are summed TIRF images of the mEos2 off-state of fixed REF cells expressing integrin β3 labeled with mEos2, growing on nanopatterned substrates with 56- or 119-nm spacing between the AuNPs. **(B)** Shown here are zoom-in PALM images corresponding to the red rectangles in **(A)**. **(C)** Given here is the result of the EMGM analysis of the PALM data shown in **(B)**. The red dots symbolize the localizations,and the blue ellipses symbolize the 2σ error ellipses of the mixture components. **(D–F)** Given here is the result of the EMGM analysis of PALM data corresponding to different REF cells (*n* = 10). The number of localizations in each mixture component is shown as a function of the area of its 2σ error ellipse, for **(D)** fibronectin-coated substrates, **(E)** nanopatterned substrates with 56-nm spacing, and **(F)** nanopatterned substrates with 119-nm spacing. The dashed white rounded rectangles in **(D,E)** are visual guides. [Figure and figure caption reproduced from (Deschout et al., 2017), figure reference omitted]. Reprinted from Biophysical Journal, 113, Hendrik Deschout, Ilia Platzman, Daniel Sage, Lely Feletti, Joachim P. Spatz, Aleksandra Radenovic, Investigating Focal Adhesion Substructures by Localization Microscopy, 2508-2518, Copyright (2017), with permission from Elsevier.

Clustering of integrins (Figure 4), with and without the help of adaptor proteins and independent of F-actin (Cluzel et al., 2005) and MII activity (Choi et al., 2008), builds the second step of NA formation. Understanding of this process is greatly facilitated by the emergence of super-resolution microscopy (SR) techniques. The latter provide optical images with spatial resolutions below the diffraction limit of light of the order of ~ 100 nm (Sigal et al., 2018). Therefore, it should be possible to resolve the dynamic nanoscale organization of NAs and the force transduction across individual components within FAs. However, quantitative investigations of NAs are still lacking. The main reason is that existing SMLM techniques require cluster analysis tools, which have been developed for relatively simple cases, such as membrane protein clusters without strong heterogeneity in size, shape, and density (Nicovich et al., 2017; Nieves and Owen, 2020). Several studies have addressed this by designing novel approaches to investigate the inner architecture of NAs and FAs, such as one based on the expectation-maximization of a Gaussian mixture (EMGM) (Deschout et al., 2017). The imagining was carried out on specifically bio-functionalized substrates, on which ordered patterns of nanoscale adhesive spots were provided (Arnold et al., 2004). Such substrates have already been used to probe the behavior of FAs on the nanoscale (Geiger et al., 2009; Schvartzman et al., 2011). In this way, the spatial organization of FA binding sites is precisely controlled, ensuring that the observed substructures are not substrate artifacts. Application of this improved EMGM method on the photoactivated localization microscopy (PALM) data showed that FAs are composed of structures with areas between 0.01 and 1 μm2, containing 10 to 100 localizations, and exhibiting strong eccentricities (Figure 5). This approach is very promising for studies of NAs, and may in future provide new insights in the cluster formation.

So far, however, various nanoscale distributions have been observed for integrins. Clusters as small as 2–3 integrins were reported using electron microscopy (Li, 2003), while clusters observed in SMLM range from tens to hundreds of molecules. Some of the first application of SR techniques yielded 100 nm large NAs, containing on average 50 integrins (Changede et al., 2015). This data is contrasted by a more recent work with improved EMGM method used on PALM data, when it was determined that FAs cover areas between 0.01 and 1 μm^2^. Using EMGM, localization uncertainties, an important and unavoidable aspect of any SMLM experiment, could be corrected, showing that the assemblies contained 10 to 100 localizations, and exhibited strong eccentricities (Deschout et al., 2017). Notably, most existing SMLM clustering methods ignored this effect, which can lead to substantial overestimation of the size of identified localization structures.

While the dynamic behavior of NAs is still an open problem, it is nevertheless clear that clusters allow for quick rebinding after bond failure (Bihr et al., 2012; Sun et al., 2019), and the control over maturation or disassembly (Schmidt et al., 2015). Furthermore, clusters could serve as platforms for rigidity sensing (Wolfenson et al., 2016), however it is still unclear which point in the process of NA assembly corresponds to the onset of signaling.

In the absence of detailed microscopy studies, even the necessary conditions for the formation of these meta-stable aggregates are unclear. Some studies report that integrin activation is indispensable for clustering (Cluzel et al., 2005), promoting the nucleation of new structures (Saltel et al., 2009). These results are contrasted by experimental findings that show both active and inactive integrin nanoclusters in FAs (Spiess et al., 2018), obtained using extended state specific antibodies that co-localized with talin, kindlin-2 and vinculin. The existence of inactive clusters could suggest the affinity for ligands in the inactive states is sufficiently large to promote nucleation of domains, although with smaller probability than in the active state. Alternatively, one could conclude that ligand binding is not necessary for clustering, although it is possible that ligand bound states preceded cluster formation.

With mobile ligands, on the other hand, Mn^2+^ activated integrins formed small adhesion domains, which significantly increased in size if integrins themselves were maintaining lateral mobility prior to establishing bonds (Smith et al., 2008). In this case, the clustering of bound integrins was mediated by the deformed membrane. The nature and magnitude of these forces could be clearly elucidated (Janeš et al., 2019), and were proposed to play an important role in the cluster nucleation and growth (Bihr et al., 2012). Given that these types of interactions are not protein specific, they should also be seen in other ligand-receptor systems. Indeed, correlations in membrane dynamics and topography with cell spreading was reported recently in several studies of cell adhesions (Perez et al., 2008; Pierres et al., 2008; Lam Hui et al., 2012), and systematically in reconstituted passive systems based on giant unilamellar vesicles (Smith and Sackmann, 2009), including those involving integrins (Goennenwein et al., 2003; Smith et al., 2008; Streicher et al., 2009). However, this mechanism remains to be directly confirmed for integrins in the cellular context.

**Figure 6:**
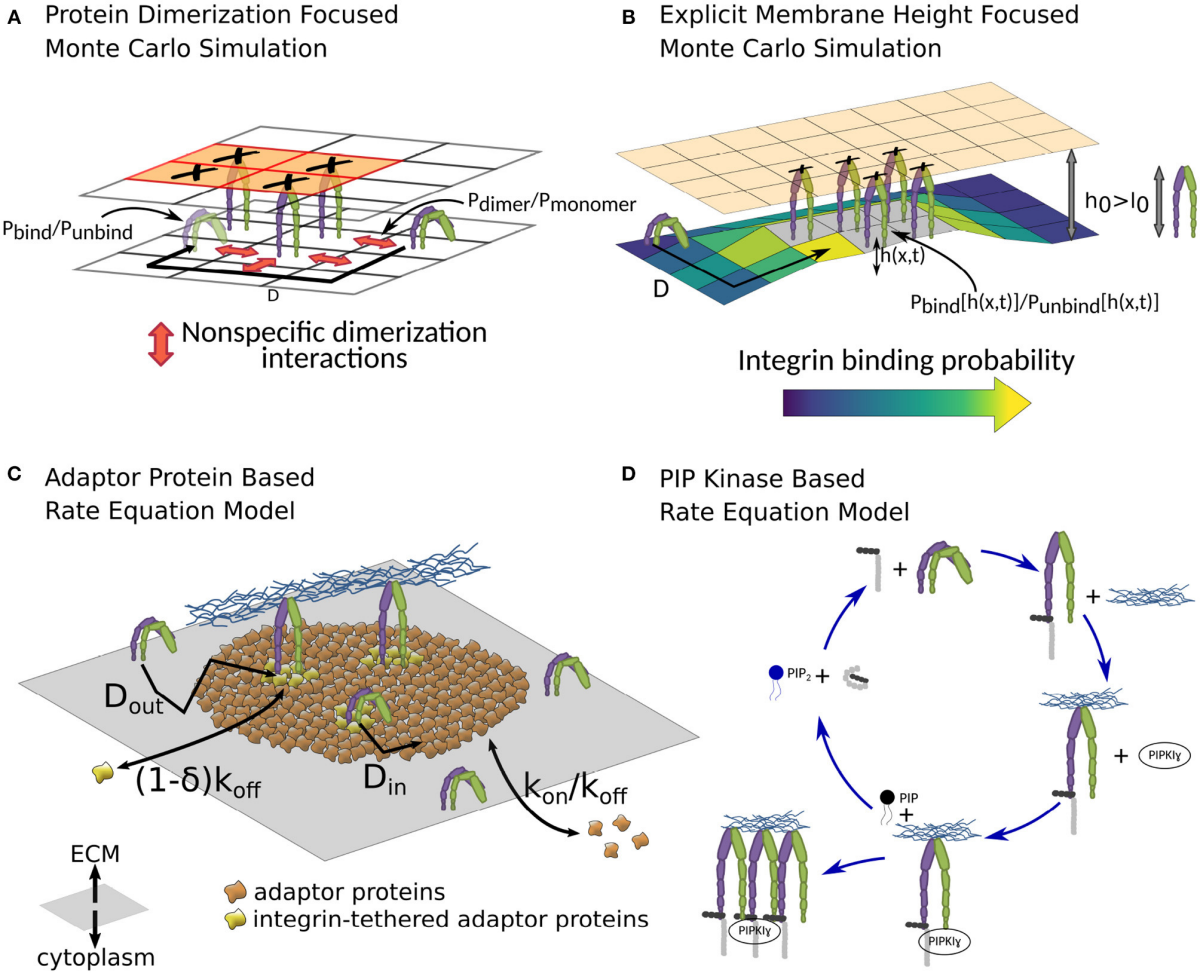
Different theoretical models of the NA formation. **(A)** In the Monte Carlo simulation by Brinkerhoff and Linderman, dimerization interactions between integrins, as well as spatial ligand distribution patterns are studied (Brinkerhoff and Linderman, 2005). They find that clustered ligand islands can increase integrin clustering, as dimerization interactions between immobile, ligand-bound integrins and mobile, unbound integrins can guide the latter toward unoccupied spots on the same ligand island. The lattice shown here is simplified compared to the one used in the publication for illustration purposes. **(B)** The coarse-grained Monte Carlo simulation in Fenz et al. (2017) allows a detailed look at one source of spatial integrin interaction, namely membrane and glycocalyx deformations (Fenz et al., 2017). Repulsions between cell and substrate, stemming among others from the shape and size of glycocalyx proteins, can result in a mismatch between the equilibrium separation of the two surfaces *h*_0_ and the combined size of integrin and ligand *l*_0_. The initial bond formation therefore has to be accompanied by a membrane deformation, imposing an additional energy cost. After initial bond formation, however, the surrounding membrane is already partly deformed, reducing the energy cost of forming additional integrin-ligand bonds, which can locally increase the rate of bond formation by several magnitudes, as well as stabilize these bonds against dissolution. **(C)** MacKay and Khadra employ rate equations to study the NA formation (MacKay and Khadra, 2019). Here the NA is defined not by their integrin content, but solely by an area of cytoplasmic adaptor proteins, which, through stochastic binding and unbinding, build the backbone of the NA, the so-called adhesion plaque. Integrins inside the plaque can bind with the surrounding adaptor proteins, which stabilizes them against unbinding, allowing a sustained or even growing NA. **(D)** In the rate equation model by Welf et al., the growth of NAs is driven by the PIP2-talin interaction. A feedback loop between PIP2 generation, subsequent release of talin autoinhibition, which, in turn, activates further integrins, can result in a sudden increase in bound integrins after the initial bond formation (Welf et al., 2012).

Most of membrane-related mechanisms include the existence of the cellular glycocalyx implicitly (Bruinsma et al., 2000; Smith and Sackmann, 2009), which was indeed found to play an important role in integrin clustering (Paszek et al., 2014). The compression and consequent expulsion of glycocalyx, by the formation of the initial bond primes the surroundings for further interactions. Concomitantly the membrane deforms toward the ligand (Janeš et al., 2019), creating a microenvironment in which the additional bonds have a much higher likelihood to form (Bihr et al., 2015; Fenz et al., 2017) (Figures 4, 6B). The tension on the bond furthermore increases their lifetimes (Kong et al., 2009), in a synergistic fashion. These effects can be further strengthened by membrane thermal (Helfrich, 1978) and active fluctuations (Turlier and Betz, 2018), which adds to the portfolio of forces acting on NAs, the latter being regulators of adhesion formation (Li and Springer, 2017; Strohmeyer et al., 2017; Oakes et al., 2018).

While the interplay between these many factors contributing to the NA formation in its early stages is not yet fully understood, there is a consensus that integrin activation increases binding to anchored, clustered ligands. Specifically, strong increase in the number of spreading cells was found for a basic pattern of 4 ligands at ~ 60 nm distance compared to 3 ligands at the same density (Schvartzman et al., 2011). If formation of NAs is seen as nucleation process, this result would suggest that the critical number of bonds to achieve a stable adhesion domain is around three, which well corresponds to theoretical predictions (Bihr et al., 2012). A similar result was confirmed by an agent-based model (Jamali et al., 2013), where large agglomerates of ligands provide the largest integrin clusters.

Simulations can also account for the competition between ligand binding and clustering different integrin types, as demonstrated on the example of β_1_ and β_3_. Closely spaced multivalent ligands promoted clusters of more than two integrins. Weak lateral intra-integrin interactions allowed transient dimer interactions with switching partners (Brinkerhoff and Linderman, 2005) but they also led to smaller number of integrins in the cluster (Bidone et al., 2019).

One scenario suggests that the link between integrin activation and clustering emerges from the lateral interactions between tails of TMDs (Li, 2003; Mehrbod and Mofrad, 2013; Ye et al., 2014). However, limited size of NAs (Changede et al., 2015; Changede and Sheetz, 2017) requires further regulation of such interactions. Moreover, the necessary activation energy between TMDs also seems too high to overcome without help (Mehrbod and Mofrad, 2013). In addition, the TMD could not drive the clustering in Mn^2+^ activated integrins, without ligands present (Cluzel et al., 2005).

Besides ligands, a number of adaptor proteins have been involved in cluster formation. The most prominent examples are kindlin (Ye et al., 2013; Changede et al., 2015) and talin (particularly its head domain) (Cluzel et al., 2005; Saltel et al., 2009; Calderwood et al., 2013; Changede et al., 2015), that have been already implicated in integrin activation (Moser et al., 2008; Zhang et al., 2008; Ye et al., 2013; Theodosiou et al., 2016). Both kindlin (Kammerer et al., 2017; Li H. et al., 2017) and talin (Golji and Mofrad, 2014) have a capacity for dimerization. For example, talin rod, which, using its integrin binding site, can rescue clustering in talin depleted cells (Changede et al., 2015). However, the efficiency of talin rod fragments was found to be inferior to the full length talin (Saltel et al., 2009). This points to a possible synergy between dimerization and binding the monovalent, and even more so for multivalent ligands with larger stoichiometries (Figures 4B, 6A), which can be perfectly well-understood on the basis of cooperative binding within the membrane.

These potentially complex stoichiometries are, however, a challenge for SMLM. Complex photo-physics of interacting fluorophores can lead to over-counting of molecules at a given location (Annibale et al., 2011a). This complicates the accurate determination of protein stoichiometries from the data. It is, however, possible to estimate the number of labeled-proteins contained in a single cluster (Annibale et al., 2011b; Baumgart et al., 2016; Spahn et al., 2016; Pike et al., 2019), but more accurate quantifications are needed and their accuracy demands further validation. A recently developed supervised machine-learning approach to cluster analysis can be an interesting candidate to cope effectively with NAs sample heterogeneity (Williamson et al., 2020). It was successfully applied on data of the C-terminal Src kinase and the adaptor PAG in primary human T cell immunological synapses (Williamson et al., 2020), but was not yet tested for talin-integrin complexation or even more generally on NA data.

Talin rod has an additional property important for the formation of NAs, namely it possesses a binding site for vinculin. Vinculin is a cytoskeletal protein with binding sites, besides talin, for actin, α-actinin, and lipids and it is usually associated with the force transmission. However, recently it was found that the talin rod domain is available to vinculin in a force-independent manner upon the release of talin autoinhibition (Dedden et al., 2019; Atherton et al., 2020). This would suggest that vinculin could play a role in NAs even before the cytoskeletal forces are involved.

The integration of vinculin could be facilitated by PI(4,5)P_2_. This phospholipid regulates talin-integrin interactions at the level of the membrane. Its association with the β_3_ units opens a binding site for the integrin on the talin head, hence controlling the talin auto-inhibition. Furthermore, the PI(4,5)P_2_ interaction with the integrin creates a salt bridge toward the membrane that prevents the close interactions of the α and β subunits. Therefore, the integrin remains in an activated, clustering-competent state (Cluzel et al., 2005; Saltel et al., 2009; Dedden et al., 2019). Consistently with these findings, sequestering of PI(4,5)P_2_ diminishes the formation of clusters (Cluzel et al., 2005).

One more protein strongly investigated in the context of integrin-clustering is α-actinin (Sun et al., 2014). It is microfilament protein necessary for the attachment of actin. Both positive and negative effects were demonstrated for β_1_ and β_3_ integrins, respectively (Roca-Cusachs et al., 2013; Shams and Mofrad, 2017), which could relate to an integrin crosstalk strategy (Bharadwaj et al., 2017). However, its role in clustering is still debated (Theodosiou et al., 2016).

This large number of molecular players involved through various interactions poses a significant challenge for comprehending the formation of NAs. A promising approach that can address this diversity is theoretical modeling based on rate equations (Zhu and Williams, 2000; Schwarz et al., 2006; Li et al., 2010; Walcott and Sun, 2010; Harland et al., 2011). An early attempt focused purely on the talin-PIP2 interaction (Figure 6D) and is therefore limited in scope (Welf et al., 2012). However, more recently, emulating the formation of entire NAs has been attempted (MacKay and Khadra, 2019). In the latter case, a higher emphasis is set on the crosslinking function of adaptor proteins (Figure 6C). The model reproduces some features found in experiments, for example the limited area of NA, even at high integrin density. It also predicts unliganded clusters. Actually, the model defines NAs as plaques of adaptor proteins and not as integrin clusters. Integrins stabilize the plaque but are not required, which allows for the possibility of preclustering adaptor proteins in the absence of integrins.

## 5. Cluster Disassembly or Maturation

Clustering of NAs into FAs or disassembly is the last step in the NA life time. The fate of NAs depends on the cell type, protein composition, and mechanical properties of the substrate (Parsons et al., 2010), as well as the attachment to the actin cytoskeleton and both MII isoforms (Vicente-Manzanares et al., 2007).

Most NAs disassemble when the lamellipodium moves past them (Choi et al., 2008) following one of several competing ways of NA disassembly, as discussed in the literature (Gardel et al., 2010). Particularly, well-studied is the role of the non-receptor tyrosine kinase FAK that is known to regulate adhesion disassembly (Webb et al., 2004), possibly through talin proteolysis (Lawson and Schlaepfer, 2012). In addition, FAK might be inhibited at the leading edge of the lamellipodium through interactions with Arp2/3, a protein complex that is related to the actin branching (Swaminathan et al., 2016). Because the regulation of rearrangements of the actin cytoskeleton is crucial for filopodia extension (He et al., 2017) and lamellipodia formation (Small et al., 2002), FAK is implicated in the spatial control of the advance of the leading edge and the NA disassembly. This regulation is facilitated by the binding of FAK to paxillin, which is also recruited by kindlin (Humphries et al., 2007; Böttcher et al., 2017; Zhu et al., 2019), to further control the adhesion turnover (Shan et al., 2009; Choi et al., 2011). Interestingly enough, vinculin, can impede the FAK-paxillin interaction (Subauste et al., 2004), while FAK may play a role in recruiting talin to NA sites (Lawson and Schlaepfer, 2012; Lawson et al., 2012), as well as Arp2/3 (Lawson and Schlaepfer, 2012). As FAK also plays an important role in signaling (Swaminathan et al., 2016), it is difficult to unravel the precise dynamical interactions in NAs.

Another simple way to dissolve NAs is by offering soluble ligands (Cluzel et al., 2005). The latter either exhibit a lateral pressure on the NA site or they compete for the integrin upon stochastic unbinding (Smith et al., 2006). Since the 3D affinity of soluble ligands is always larger than the 2D affinity of surface-confined ligands, the cluster becomes unstable.

Alternative to disassembling is the sequential maturation of NAs into FAs, and in some cases, to centrally positioned fibrillar adhesions enriched in tensin and integrin α5β1 (Iwamoto and Calderwood, 2015). The inhomogeneous structure of these assemblies were observed already over a decade ago using PALM for imaging FA proteins (Betzig et al., 2006). Just 1 year later, Shroff et al. used dual-color PALM to determine the ultrastructural relationship between different pairs of FA proteins (Shroff et al., 2007). The consensus today is that integrins bind via talin and other adaptor proteins with the actin cytoskeleton, allowing MII generated forces to act on the clusters (Yu et al., 2011). Under force the bonds strengthen, and tilt in the direction of the retrograde actin flow (Nordenfelt et al., 2017). The reinforcement of integrin assemblies is further promoted by the recruitment of vinculin (Huang et al., 2017), the crosslinking by myosins (Burridge and Guilluy, 2016), and the exposure of force-dependent cryptic binding sites (Ciobanasu et al., 2014; Yao et al., 2015, 2016) that allow for the attachment of other adhesome proteins. Finally, through mature adhesions, force is propagated from the ECM to the actin cytoskeleton over the unfolding talin that permits vinculin binding (Asaro et al., 2019), resulting in a strong signaling cascade and mechanoresponse along the adhesome, that regulates a number of physiological processes in cells.

## 6. Challenges and Perspectives in the Field of Nascent Adhesions

As presented in the above discussion, many molecular players contributing to the formation of NAs have been identified, and their mutual interplay have been established, although further investigation of specific interactions is necessary. For example, very little is known about the crosstalk between integrins of the same and different types, recently reported for early adhesions (Bharadwaj et al., 2017; Strohmeyer et al., 2017; Diaz et al., 2020; Samaržija et al., 2020). However, new research avenues in studying molecular interactions in NAs can emerge only from advances in the development of robust quantitative colocalization analysis (Levet et al., 2019). This needs to be accompanied by progress in genome editing and novel protein labeling strategies that could enable quantitative SR. So far, only very few colocalizations of active integrins with talin and kindlin (Spiess et al., 2018) and vinculin and talin (Xu et al., 2018) could be observed. This can be either a technical issue, associated with protein expression, probe photophysics, and the limited choice of labeling pairs and fluorophores. It could also point to unknown integrin regulators (Spiess et al., 2018), and hidden interactions that remain to be revealed. For this purpose, molecular dynamics simulations will become increasingly important as they provide unmatched details in competing binding interactions (e.g., Mehrbod and Mofrad, 2013). With appropriate level of coarse-graining, larger complexes and slower structural changes are coming within reach of molecular dynamics simulations, which now can explicitly address integrin activation and clustering.

Probably, however, the most acute issue is the spatiotemporal evolution of NAs and the role of complex stoichiometries. Namely, the dynamics of NAs is subject of intense debate as the constitutive clusters could be either stationary or showing stochastic transient immobilization (Spiess et al., 2018). This problem is very closely related to the sensory capacity of NAs and the onset of signaling, that are equally understood. Resolution of these open questions requires new techniques that can deal with the fast molecular turnover within NAs. However, this is still a significant challenge for the single molecule localization microscopy (Orré et al., 2019) such as PALM (Betzig et al., 2006), stochastic optical reconstruction microscopy (Rust et al., 2006) or super-resolution optical fluctuation imaging (Dertinger et al., 2009). First promising insights into the dynamics of NAs nevertheless were provided by the single particle tracking PALM (Inavalli et al., 2019), which revealed integrin cycling between free diffusion and immobilization, while transient interaction with talins promoted integrin activation and immobilization (Rossier et al., 2012). Further studies of integrin dynamics will require techniques such that can operate at micro second-time scales with 1 nm precision of molecules located few nanometers apart. An example of such a method is minimal photon fluxes nanoscopy (Balzarotti et al., 2017), although other approaches are starting to appear and will need to be employed in the research of NAs.

Another challenge in studies of NAs is the impact of force. Although not strictly related to actomyosin activity, forces on integrin complexes arise due to the spacial confinement of the molecular players and result in load-dependent competition for binding partners. Different sources of forces may play a role in NA formation, prior to their maturation into FAs. Specifically, the glycocalyx and the membrane are anticipated to generate relatively strong tensions and direct stochastic forces on the individual integrins and the clusters (Paszek et al., 2014; Li and Springer, 2017; Strohmeyer et al., 2017; Sengupta and Smith, 2018). The understanding of these effects relies on the development of force sensors (Tan et al., 2020), and techniques which combine the force application with SR. Furthermore, given the intrinsically non-equilibrium and noisy setting, theoretical support in formulating and validating the appropriate hypothesis on the role of confinement is necessary.

A particularly useful tool in the research of integrin adhesion so far have been functionalized substrates (Goennenwein et al., 2003; Schvartzman et al., 2011; Liu et al., 2014; Changede et al., 2019). Manipulation of stiffness, spatial coordination and mobility of binders allowed to provide mechanical cues which could be exploited to resolve the response of different cell models. Based on this long standing success, it is expected that patterned substrate will continue to play an important role in studies of NAs. Especially interesting should be their combination with specifically designed cell models that express different types of integrins on the plasma membrane surface.

Furthermore, these substrates could be very successfully combined with reconstituted systems. The latter serve as an ideal bridge between the biological complexity and theoretical modeling. Reconstituted systems, typically based on giant lipid vesicles, were instrumental in elucidating the role of mechanical properties of binders, as the role of the receptor and ligand density and mobility in the cell recognition process (Smith and Sackmann, 2009). Furthermore, vesicle-substrate adhesion was successfully used to study the physical mechanisms that regulate ligand-receptor binding, including the role of stochastic membrane deformations, fluctuations and composition, as well the steric repulsion role of the glycocalyx (Sengupta and Smith, 2018). However, the simplicity of these assemblies may not represent the appropriate biological complexity of NAs. To circumvent that issue, more recently droplet-stabilized giant unilamellar vesicles were designed that can be sequentially loaded with talin and kindlin (Weiss et al., 2018). These systems show great potential for the studies of NAs, and could be used to drive the development and validation of theoretical models and simulations used to describe the growth process.

At the current stage, most theoretical models that attempt to capture the formation of NAs account for the molecular complexity of the system, but capture the bio-mechanical context only implicitly, if at all. There is also another class of models that is capable of resolving the stochastic nature of NA formation and the forces acting on the bonds with relatively high level of detail, but they are nearly void of molecular information. Future efforts are likely to bring closer these two distinct families of approaches, with the aim of providing a more reliable foundation that is required to capture the development of NAs in a predictive manner.

Finally, it is a hope that the lessons learned in the studies of NAs may be useful in the context of other integrin-based structures. For example, in hemidesmosomes, linked to the internal keratin intermediate filament network, α6β4 integrins mediate adhesion of epithelial cells to the underlying basement membrane (Walko et al., 2015). In reticular adhesions, which serve to maintain the attachment of cells to the extracellular matrix during mitotic rounding and division, α*Vβ*5 integrins, clathrin and endocytic adaptors also form adhesive complexes (Grove et al., 2014; Elkhatib et al., 2017; Leyton-Puig et al., 2017; Lock et al., 2018, 2019). Currently, it is not known whether these different types of adhesion have precursor structures analogous to NAs. However, it is highly likely that tools, methods and approaches developed in studies of NAs may prove to be useful in these potentially different settings.

In closing, we strongly believe that joint advances in SR, the development of model systems and technology for manipulations of proteins, as well as theoretical approaches are required to further propel our understanding in molecular mechanisms of integrin organization, stoichiometry and dynamics at the nanoscale. This will not only allow us to rationalize the observed phenomena, but also gain important concepts and tools that can be used to resolve the physiological role of integrin based structures, but can be further applied beyond the NA research.

Integrin involvement in pathological conditions is mostly the consequence of changes in the expression, either up- or down-regulation. Prominent examples here are tumorigenesis but also the response to chemo- or radiotherapy (Cooper and Giancotti, 2019). Therefore, integrin repertoire changes are an active target for drug development in tumors with the potential to inhibit metastasis, as well as to overcame resistance to chemotherapy or radiotherapy. However, despite convincing experimental evidence that demonstrates the capacity of integrin inhibitors and monoclonal antibodies to contribute to inhibition of cancer progression, metastasis, or boost therapeutic effects, no integrin-targeting drugs have been registered as anti-cancer drug (Desgrosellier and Cheresh, 2010; Seguin et al., 2015; Dickreuter and Cordes, 2017; Hamidi and Ivaska, 2018; Alday-Parejo et al., 2019; Cooper and Giancotti, 2019). Integrins are, nonetheless, used as targets in the prevention of blood clots during the opening of blood vessels in the heart (Tam et al., 1998), multiple sclerosis (Polman et al., 2006) and Crohn's disease (Gordon et al., 2001; Rosario et al., 2017). Furthermore, since the accumulation of disorganized ECM is modulated by several integrin heterodimers via activation of latent transforming growth factor-β, the selected integrins are considered as promising therapeutic targets for fibrosis (Kim et al., 2018). Besides integrin up- or down-regulation, integrin mutations are also associated with some diseases like junctional epidermolysis bullosa, caused by mutations in either integrin subunit of integrin α6β4 forming hemidesmosomes or integrin α3, which pairs with β1, forming FAs (McGrath, 2015; Walko et al., 2015). Furthermore, integrin related diseases may also be caused by impaired activation as observed on platelets and leukocytes (Alon and Etzioni, 2003). Integrins are also involved in bacterial (Hoffmann et al., 2011) and viral infections, either in attachment or internalization (Hussein et al., 2015), thus representing possible target molecules to combat infectious diseases.

## Author Contributions

BH conceived and wrote the first draft of the manuscript under the supervision of A-SS. All authors contributed to the writing of the final paper.

## Conflict of Interest

The authors declare that the research was conducted in the absence of any commercial or financial relationships that could be construed as a potential conflict of interest.
